# Diversity of entomopathogenic fungi associated with Mediterranean fruit fly (*Ceratitis capitata *(Diptera: Tephritidae)) in Moroccan Argan forests and nearby area: impact of soil factors on their distribution

**DOI:** 10.1186/s12898-020-00334-2

**Published:** 2020-11-24

**Authors:** Ayoub Hallouti, Mohamed Ait Hamza, Abdelaziz Zahidi, Rachid Ait Hammou, Rachid Bouharroud, Abdellah Ait Ben Aoumar, Hassan Boubaker

**Affiliations:** 1 Laboratoire de Biotechnologies Microbiennes et Protection des Végétaux, Faculté des Sciences, Université Ibn Zohr, BP 8106 Agadir, Morocco; 2 Laboratoire de Biotechnologies et Valorisation des Ressources Naturelles, Faculté des Sciences, Université Ibn Zohr, BP 8106 Agadir, Morocco; 3 Unité de Production Intégrée des Cultures, INRA, Agadir, Morocco

**Keywords:** Entomopathogenic fungi, Communities, Soil ecology, *Ceratitis capitata*, Biological control

## Abstract

**Background:**

Studying the ecology of biocontrol-agents is a prerequisite to effectively control medfly (*Ceratitis capitata* (Diptera: Tephritidae)) with entomopathogenic fungi. In this context, factors affecting the occurrence and distribution of medfly-associated entomopathogenic-fungi were studied. Soil samples (22) were collected from natural and cultivated areas of Souss-region Morocco.

**Results:**

A total of 260 fungal isolates belonging to 22 species and 10 genera were obtained by using medfly pupae as bait. Medfly-associated fungi were detected in all studied soils and pupae infection percentages ranged from 3.33% to 48%. Two genera, *Fusarium* and *Beauveria* were the most frequent with 83 isolates (32%) and 50 isolates (19.23%) respectively. Pathogenicity test of isolated species against medfly pupae showed high mortality rates up to 91% for some strains. Principal component analysis (PCA) demonstrated a strong influence of origin, physical, and chemical properties of soil on the abundance of these fungi. In general, medfly-associated fungi were more abundant in soils with moderate pH (7.5 to 8) having high sand and organic content. High relative humidity negatively influenced the abundance of these fungi. Both factors directly affected the fungal infection percentages in pupae. The response of fungi to these parameters varied among species. According to principal component analysis (PCA), the soils of argan fields and forests were more suitable for the development of medfly-associated fungi than citrus orchards.

**Conclusions:**

These results guide identifying suitable soils for the effective application of entomopathogenic fungi as biological control agents. In summary, isolated indigenous strains seem to be a promising option to control *C. capitata*.

## Introduction

Soil is the natural habitat for entomopathogenic fungi which plays an essential role in regulating the populations of soil-inhabiting insects. Mediterranean fruit fly (*Ceratitis capitata* Wiedemann (Diptera: Tephritidae)) or medfly is one of the most destructive fruit pests in Morocco and several parts of the world such as Europe, South America, North America, and Asia [1–3]. Traditionally, the control of *C. capitata* populations is often based on chemical insecticides, which are known for toxicity to the environment and human health [4, 5]. Therefore, the use of entomopathogenic fungi provides a promising bio-control alternative.

To effectively control *C. capitata* by using entomopathogenic fungi (EPF), identification and selection of indigenous fungi strains are necessary. Except for the studies of Imoulan et al. [6] and Imoulan and Elmeziane [7], the tested entomopathogenic fungi strains against *C. capitata* have never been isolated from medfly infected individuals or soils containing larvae and pupae of this insect. The introduction of non-indigenous entomopathogenic strains can reduce the effectiveness of biocontrol agents and pose ecological risks [8–10]. Therefore, isolation of medfly-associated entomopathogenic fungi must be carried out from its natural environment. The selection of indigenous fungi strains increases the probability of an effective control [10, 11]. Besides, if applied as bio-pesticides, these strains can overcome environmental stress by improved adaptation to environmental conditions [6, 12]. Several studies have reported that the tolerance of entomopathogenic fungi to climatic conditions is strongly related to its natural habitat [6, 13, 14].

Soils of Argan (*Argania spinosa* (L.) Skeels (Ericales: Sapotaceae)) forests and citrus orchards in the Souss region are known as a natural refuge for medfly and thus are optimum locations to search for medfly-associated entomopathogenic fungi. These soils are the natural habitat of *C. capitata* L3 larvae and pupae [15, 16]. Imoulan et al. [6] has isolated more than 118 isolates of *Beauveria bassiana* ((Bals.-Criv.) Vuill.; Hypocreales: Cordycipitaceae) and many strains of *Verticillium lecanii* ((Zimm) Viégas; Hypocreales: Cordycipitaceae) from argan soils. These isolates showed significant pathogenicity against *C. capitata* larvae and pupae during laboratory experiments [6, 7]. Moreover, its pupation in soil offers an opportunity to develop an effective control strategy against this pest. Nevertheless, this opportunity has never been thoroughly explored.

Soil provides a nutritious environment for entomopathogenic fungi and protection against climatic conditions. However, the parameters of this medium such as texture, pH, electrical conductivity, relative humidity, carbon/nitrogen ratio, and organic matter content directly affect the availability and abundance of fungal species [17–19]. Several studies have reported the effect of soil type, climatic factors, and agricultural practices on the distribution of entomopathogenic fungi [20–22]. Thus, suitable levels of these soil parameters may promote the development of specific species than others. Quesada-Moraga et al. [19] demonstrated that soil organic matter content significantly affects cation-exchange capacity (CEC), which influences water absorption and fungal spore germination. Moreover, they found that *Metarhizium anisopliae* ((Metsch) Sorokin; Hypocreales: Clavicipitaceae) strains prefer soils with a pH below 7 whereas *Beauveria bassiana* strains adapt better to basic soils. The water availability and soil texture have also been reported to directly affect the vertical movements, availability, and survival of the conidia of *B. bassiana, M. anisopliae* and *V. lecanii* [10, 18].

The present study was aimed to isolate and identify the medfly-associated entomopathogenic fungi. The isolation was carried out from three types of soils: soils of argan forests, cultivated fields containing argan trees, and citrus orchards of the Souss region, Morocco. The physical and chemical properties of these soils were studied to understand their relationship with the abundance and distribution of entomopathogenic fungal strains as well as the ecology of the fungal communities.

## Materials and Methods

### Insects

*C. capitata* larvae were collected from infested argan fruits and reared in the laboratory under controlled temperature (25 ± 2 °C) and photoperiod (14 h/10 h, L/D). Medfly adults were provided with water and a sugar-yeast nutrient medium (¾ sucrose + ¼ yeast extract). The larval medium consisted of 940 g wheat bran, 50 g yeast extract, 5 g Nipagine, and 5 g glucose in 1000 ml distilled water [16]. *C. capitata* pupae were used as EPF baits in trapping bait test and pathogenicity test.

### Soil samples

**Fig. 1:**
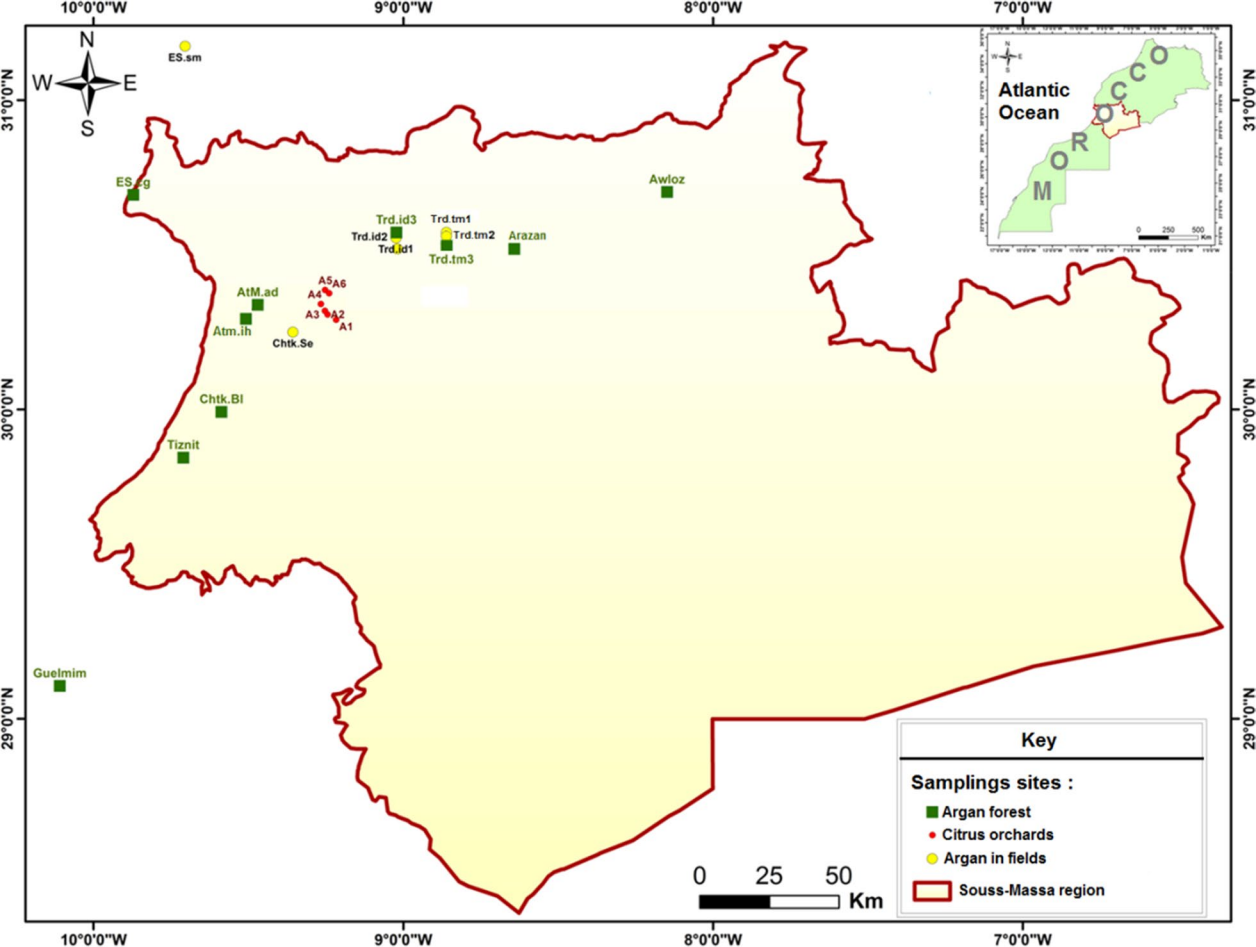
Sampling sites: location within the Moroccan territory and Souss-Massa region (the map was produced using ArcMap V10.0 software)

Soil samples were collected from Souss Argan forests and nearby areas (Fig. 1). The soil of the argan sub-tree is the natural habitat of *C. capitata* L3 larvae and pupae [6, 15, 16]. Therefore, choosing this soil increases the chance of trapping entomopathogenic fungi isolates with greater virulence against C. *capitata* compared to other fungal species.

Soil samples containing three subsamples were collected from twenty-two different sites. For each subsample, 1 kg soil was collected from the sub-tree area of about ten trees. Samples were taken at a depth of 10–20 cm after removing surface litter [20]. Soil obtained for the subsamples was mixed to obtain a homogeneous sample representing the sampling site. Sampling sites were selected to represent the variability of soil types, soil origin, and climate (Fig. 1). To study the effect of the soil’s origin on the availability of EPF, soil samples were collected from argan forest (natural area), argan fields (intercropping plants), and citrus orchards (conventional crops).

Samples were placed in plastic bags to prevent water loss and, immediately transferred to the laboratory to store at 4 °C in dark [20]. Each bag was provided with a unique reference code to identify the sampling site. To differentiate between soil samples, an analysis was performed to study the physical and chemical properties such as texture (sand, silt, and clay), pH, electrical conductivity (EC), humidity (RH), organic matter content (OM), and carbon/nitrogen ratio (C/N). The pH, relative humidity, and electrical conductivity of these samples were immediately measured in the laboratory [23].

### Trapping of entomopathogenic fungi

**Fig. 2:**
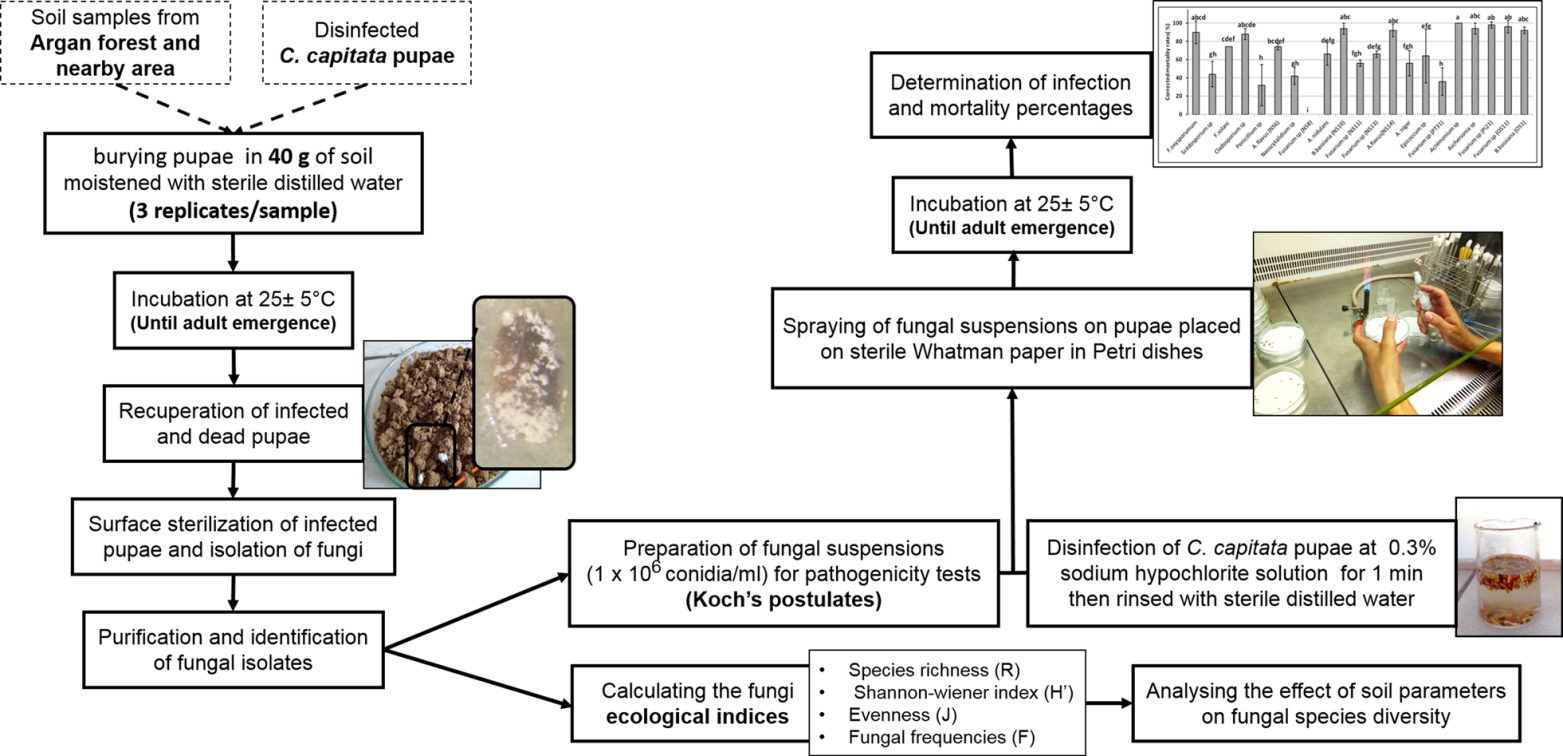
Illustration of the experiments conducted to study the diversity of *C.capitata* associated fungi in Argan forest soil and nearby area and the pathogenicity of these fungal isolates against this pest pupae

*Ceratitis capitata* associated entomopathogenic fungi were isolated by following the bait method [24]. Medfly pupae were used as a bait to trap medfly specific EPF strains (Fig. 2). To our knowledge, this is the first report of using medfly pupae as bait for the isolation of entomopathogenic fungi.

To prepare the baits, *C. capitata* pupae were disinfected for 1 min in a solution of distilled water containing 0.3% sodium hypochlorite and then rinsed with sterile distilled water [25]. Twenty pupae were buried in a petri dish containing 40 g of soil moistened with sterile distilled water [26]. Petri dishes were sealed with parafilm and incubated at 25 °C for 10 days. To obtain reliable data and to increase the chances of trapping EPF, previous studies recommend using more than 5 individuals of bait insects [19, 27]. Three replicates were prepared from each soil sample and a total of 60 pupae were used.

Pupae that were unable to go to adult emergence were collected. These pupae were surface-sterilized then placed in sterile Petri dishes containing moistened Whatman paper with sterile distilled water and incubated at 25 °C [28] (Fig. 2). Towards the end of this second incubation, the fungal infection percentage of pupae was calculated after determining the number of individuals (cadavers) representing fungal infection "N". The infection percentage (Ir) was determined according to the following formula:

$$\mathrm{Ir}\left(\mathrm{\%}\right)=\frac{\mathrm{N}}{60}\mathrm{x}$$ 100.

### Isolation and identification of fungi

Pupae with external mycelial growth of fungi were disinfected and placed directly in Petri dishes containing PDA (potato dextrose agar) supplemented with chloramphenicol (0.25 g/l) [29, 30]. To eliminate saprophytic microbial flora, dead pupae were disinfected for 1 min with a solution of distilled water containing 0.3% sodium hypochlorite and rinsed five times with sterile distilled water [25]. Petri dishes were incubated at 25 °C for 3 to 5 days. The colonies around pupae were purified through successive subcultures on PDA medium. Fungal isolates were identified based on macroscopic and microscopic criteria by following specific taxonomic keys [31, 32] (Additional file 1).

### Fungi ecological indices

The diversity parameters such as species richness (R), Shannon–wiener index (H’), evenness of fungal communities (J), and frequencies (F) were estimated based on Mo et al. [33] and Magurran [34] methods (Fig. 2). The species richness was calculated according to the formula $R=\left(S-1\right)/Ln(N)$, where S is the number of species and N is the total number of isolates. Shannon index (H’) was measured by the formula ${H}^{^{\prime}}=\sum_{i=1}^{n}\frac{Xi}{N}Ln (\frac{Xi}{N})$ [33], where Xi is the number of observations of “i” species and N is the total number of isolates observed in each sample. The evenness (J) of fungal communities was represented by J = H’/H max. The occurrence frequencies (F) of each species were calculated as follows $\mathrm{F}=\left(\frac{\mathrm{individual\,number\, of\, a\, species}}{\mathrm{ndividual\, number\, of\, all\, species}}\right)\mathrm{x}100$.

### Preliminary pathogenicity test (Koch’s postulates)

After the purification of fungal isolates, 22 potential entomopathogen strains were selected. A preliminary test was performed to check their pathogenicity on *Ceratitis capitata* pupae (Fig. 2). Disinfected medfly pupae were placed on Whatman paper in Petri dishes and inoculated by spraying 2 ml of fungal suspension at a concentration of 1 × 10^6^ conidia/ml. This concentration of EPF has already been reported as effective in previous studies [35–37]. Twenty pupae per replicate were sprayed with fungal suspension and three replicates (n = 60) were prepared for each strain. The control pupae were sprayed with sterile distilled water containing 0.1% of Tween 80. Treated pupae were maintained at a temperature of 25 °C. The infection percentage was determined under a binocular loupe (40 × magnification) after 24 h of inoculation and every 48 h later on. To prevent horizontal transmission of the pathogen (EPF) between treated pupae, infected individuals were regularly removed. Towards the end of the test, re-isolation of the entomopathogens was carried out from pupae to confirm that the tested fungi have caused the observed mortality (Koch’s postulates).

To eliminate the natural mortality of the insect, mortality rates were corrected using Abbott’s formula [38] $\mathrm{CM}\left(\mathrm{\%}\right)=\frac{\left(\mathrm{Mt}-\mathrm{Mc}\right)}{\left(100-\mathrm{Mc}\right)} \times 100$ where Mt is the mortality rate in the treatment and Mc represents the average of mortality rates in control.

### Data analysis

Statistical analyses were performed in open source “R” software [39, 40]. Principal component analysis (PCA) of soil parameters, the abundance of medfly-associated fungi in the soil, and co-inertia between the abundance of genera and soil parameters were performed by using "Factoextra" [41] and "Factominer" [42] packages. The circles of correlations were also obtained by using "Psy" [43] and "Corrplot" [44] packages. One-way ANOVA and Fisher's LSD tests were carried out in Statistica (V6.0) software to compare isolates according to mortality rates [45].

## Results

### Occurrence and pathogenicity of medfly-associated fungi

#### Occurrence frequencies

During this study, 1320 *Ceratitis capitata* pupae were used as bait to trap and isolate entomopathogenic fungi from 22 soil samples. Approximately 23% (300) pupae were fungi infected. Results demonstrated that all studied soil samples contain medfly-associated fungi with infection percentages ranged from 3.33 to 48%. The isolation of these fungi on PDA yielded 260 fungal isolates belonging to 22 species and 10 genera. Further, the abundance and richness of fungal species vary according to soil samples.

**Fig. 3:**
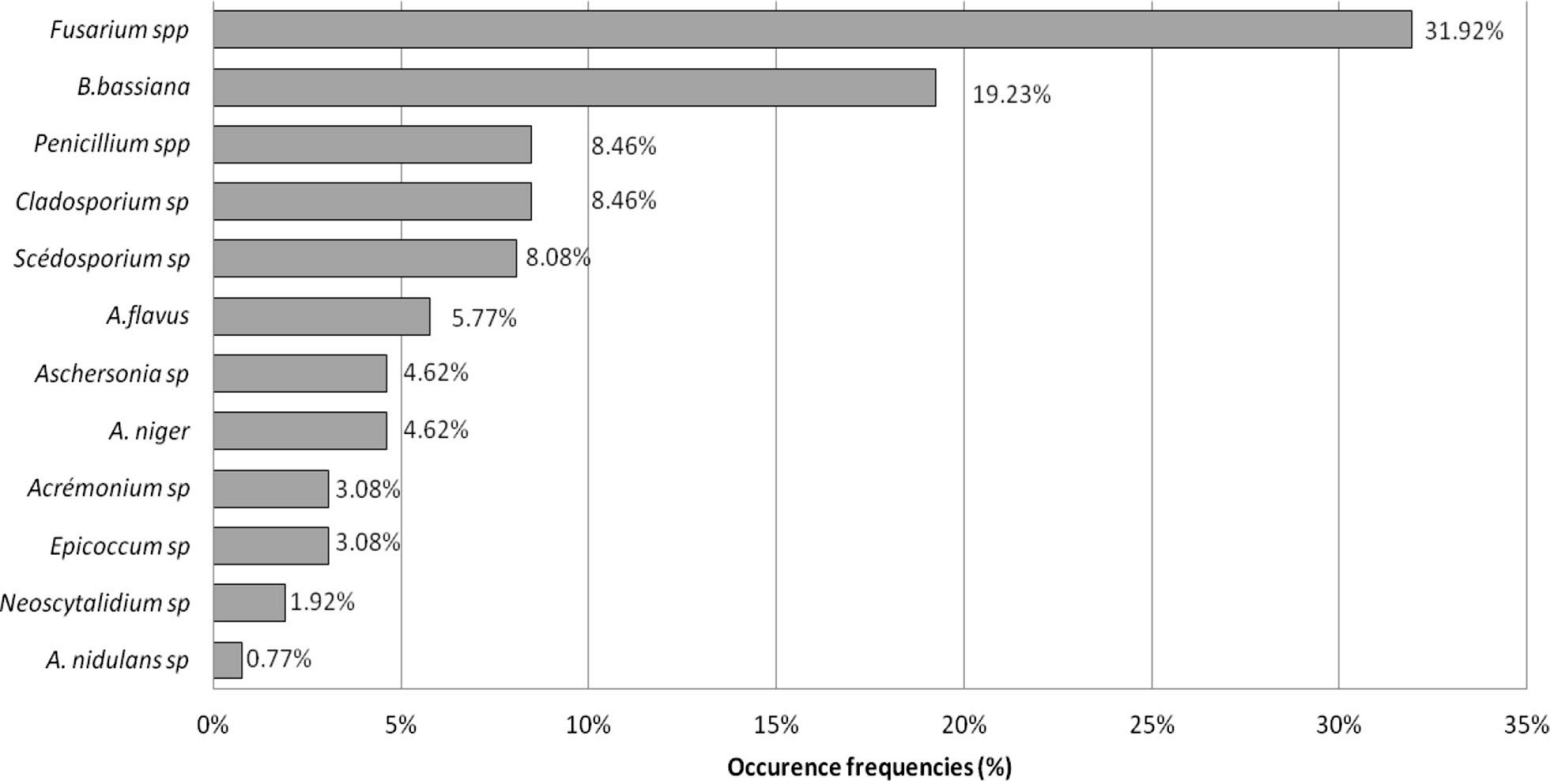
Occurrence frequencies of isolated genera calculated from the number of the occurrence of a species/ number of the occurrence of all the species

The occurrence frequencies of fungal species varied as illustrated in Fig. 3. *Fusarium* (Link; Hypocreales: Nectriaceae) (Additional file 1: Fig. S1) was the most frequent genus in the studied soils and accounted for 32% (83 isolates) followed by *Beauveria bassiana* species (Additional file 1: Fig. S2) with 19% (50 isolates) and *Scedosporium* sp. (Sacc. ex Castell. & Chalm.; Microascales: Microascaceae) (Additional file 1: Fig. S8), *Penicillium* sp. (Link; Eurotiales: Aspergillaceae) (Additional file 1: Fig. S9) and *Cladosporium* sp. (Link; Capnodiales: Cladosporiaceae) (Additional file 1: Fig. S10) with more than 8% isolates (22 isolates for each). The occurrence frequencies of *Aschersonia* sp. (Mont.; Hypocreales: Clavicipitaceae) (Additional file 1: Fig. S5), *Aspergillus flavus* (Link; Eurotiales, Aspergillaceae) (Additional file 1: Fig. S6), and *Aspergillus niger* (Tieghem; Eurotiales: Aspergillaceae) (Additional file 1: Fig. S7) strains were ranged from 4.6% to 5.7%. On the other hand, results showed that *Acremonium* sp. Link (Hypocreales) (Additional file 1: Fig. S3), *Epicoccum* sp. (Link; Pleosporales: Didymellaceae) (Additional file 1: Fig. S4), *Neoscytalidium* sp. (Crous & Slippers; Botryosphaeriales: Botryosphaeriaceae), and *Aspergillus nidulans* ((Eidam) G. Winter; Eurotiales, Aspergillaceae) were less frequent in argan and citrus soils with less than 3.1% occurrence frequency. Among isolated strains, the genus *Fusarium* represented a higher diversity with seven different strains.

#### Pathogenicity tests

**Fig. 4:**
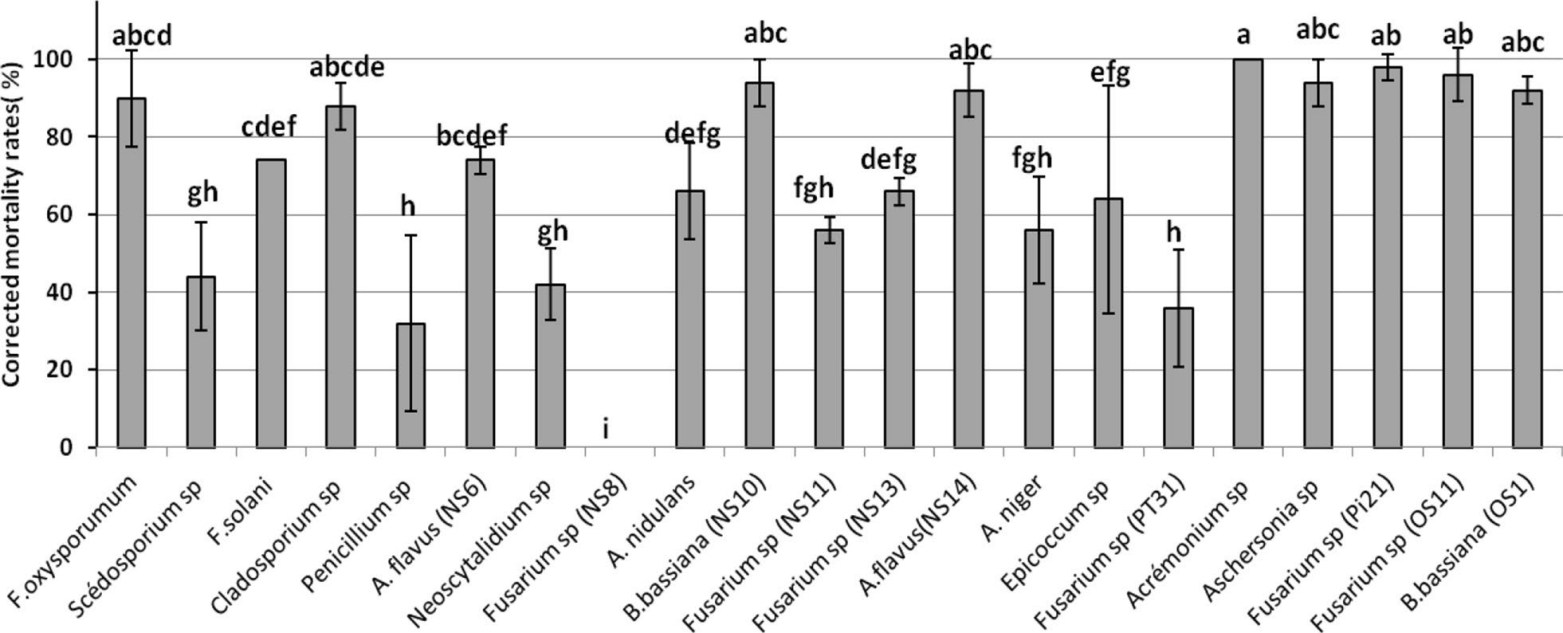
Corrected mortality rates of medfly pupae related to the EPF strains calculated using Abbott [38] formula.—Mean (± SE) of corrected mortality, Means followed by different letters differ significantly (comparison were performed using Fisher-LSD test, P < 0.05), Treatments sharing the same letter are not significantly different

To confirm the pathogenicity, a virulence test of 22 fungal isolates was carried out (Fig. 4). Analysis of the results showed highly significant differences between the isolates (p-value = 0.0000 – F_isolate_ = 7.994 − df = 21). Determination of homogeneous groups by Fisher’s LSD demonstrated that *Acremonium* sp. was the most virulent strain and caused 100% pupae mortality. Strains of the second group consisted of *Fusarium* sp. (Pi21), *Fusarium* sp. (OS11), *B. bassiana* (NS10), *Aschersonia* sp. (Pt14), *B. bassiana* (OS1), and *A. flavus* with corrected mortality rates up to 91% followed by 89.99% mortality by *Fusarium oxysporum* (Schltdl.) (NS1) and 87.99% mortality by *Cladosporium* sp. These strains formed homogeneous groups as “a”, “ab”, “abc”, “abcd” and “abcde”, respectively with mortality rates over 87%.

On the contrary, strains of *Scedosporium* sp, P*enicillium* sp., *Neoscytalidium* sp. NS7, *A. niger,* and *Fusarium* spp. (NS11 and Pt31) caused 55% pupae mortality whereas *Fusarium* sp. (NS8) did not affect medfly pupae (0% mortality).

### Influence of soil parameters on the abundance of medfly-associated fungi

**Fig. 5:**
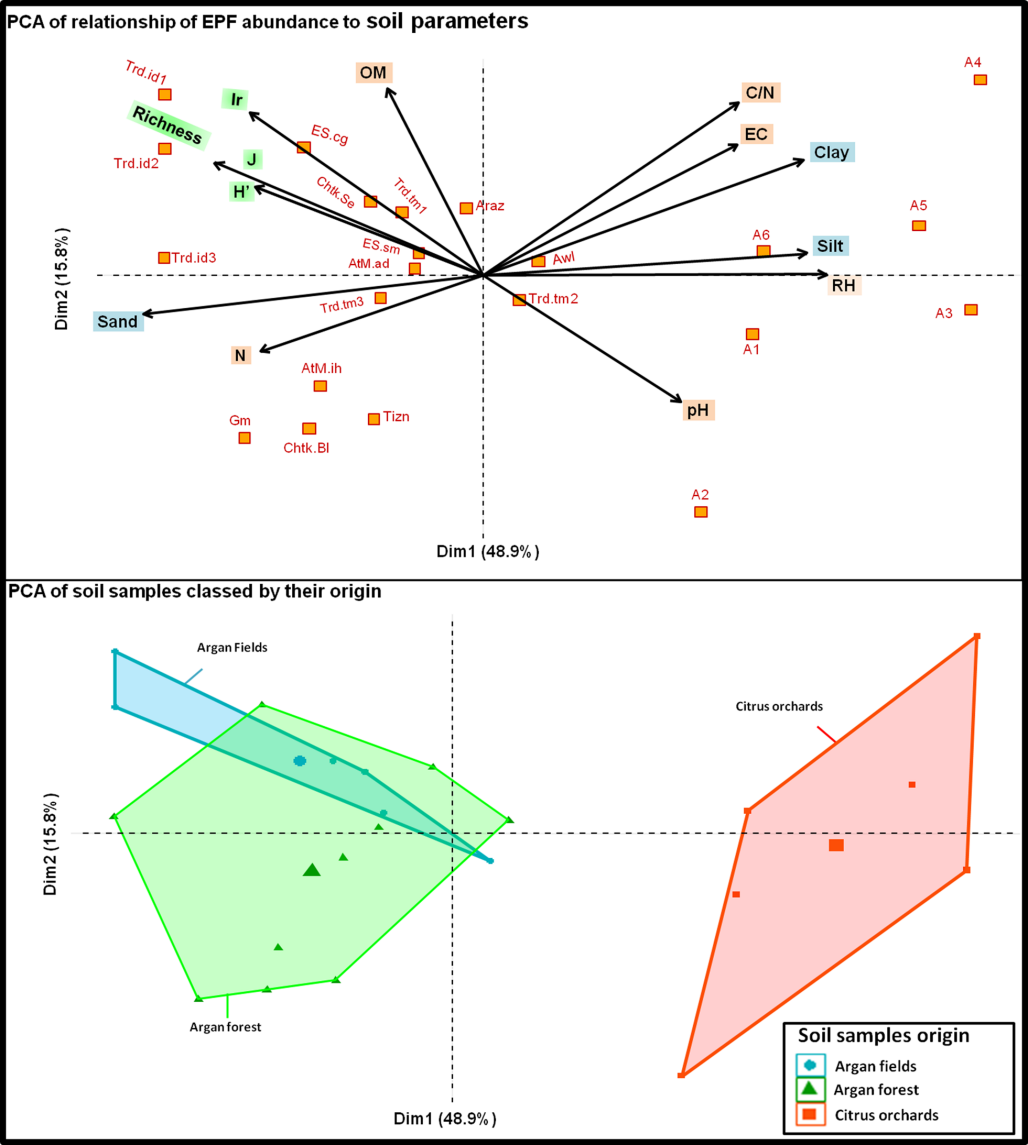
PCA of the relationship between soil parameters, soil origin to the abundance and diversity of medfly-associated entomopathogenic fungi

Principal component analysis (PCA) results (Fig. 5) showed the effect of soil parameters on the abundance of medfly-associated entomopathogenic fungi.

Results revealed significant differences between soil samples with a general variability of 64.7%. The first axis of PCA (Dim1) represents 48.9% variability related to soil texture (sand and silt content), relative humidity and nitrogen content (N), Shannon index, evenness, and generic richness. The second axis of PCA (Dim 2) represents a 15.8% variability that is strongly related to pH, organic matter content, infection percentages, and C/N ratio. Distribution of these parameters revealed that the generic richness and ecological balance of these soils increase the chances of fungal infection in insects. Likewise, high sand content in the soil promotes the infection process. The abundance of entomopathogenic fungi in soils also requires high organic matter content and moderate pH (around 8). Moreover, our results showed that high relative soil humidity negatively influences EPF’s abundance and insect infection percentages (Ir). C/N ratio is an indicator of soil health and is generally related to microbial activity and abundance of fungi in the soils.

PCA demonstrated that soils of argan fields (argan with crops) and soils of argan forests were more suitable for the development of medfly-associated entomopathogenic fungi. Moderate pH, high organic matter content, adequate moisture, and sandy texture make these soils a good habitat for medfly entomopathogenic fungi. However, citrus soils may contain pesticide residues that prevent the growth of microorganisms and possess a high C/N ratio, pH, and EC. Besides their silty texture (Fig. 5), irrigation in citrus orchards increases the soil relative humidity which influences the development of entomopathogenic fungi.

**Fig. 6:**
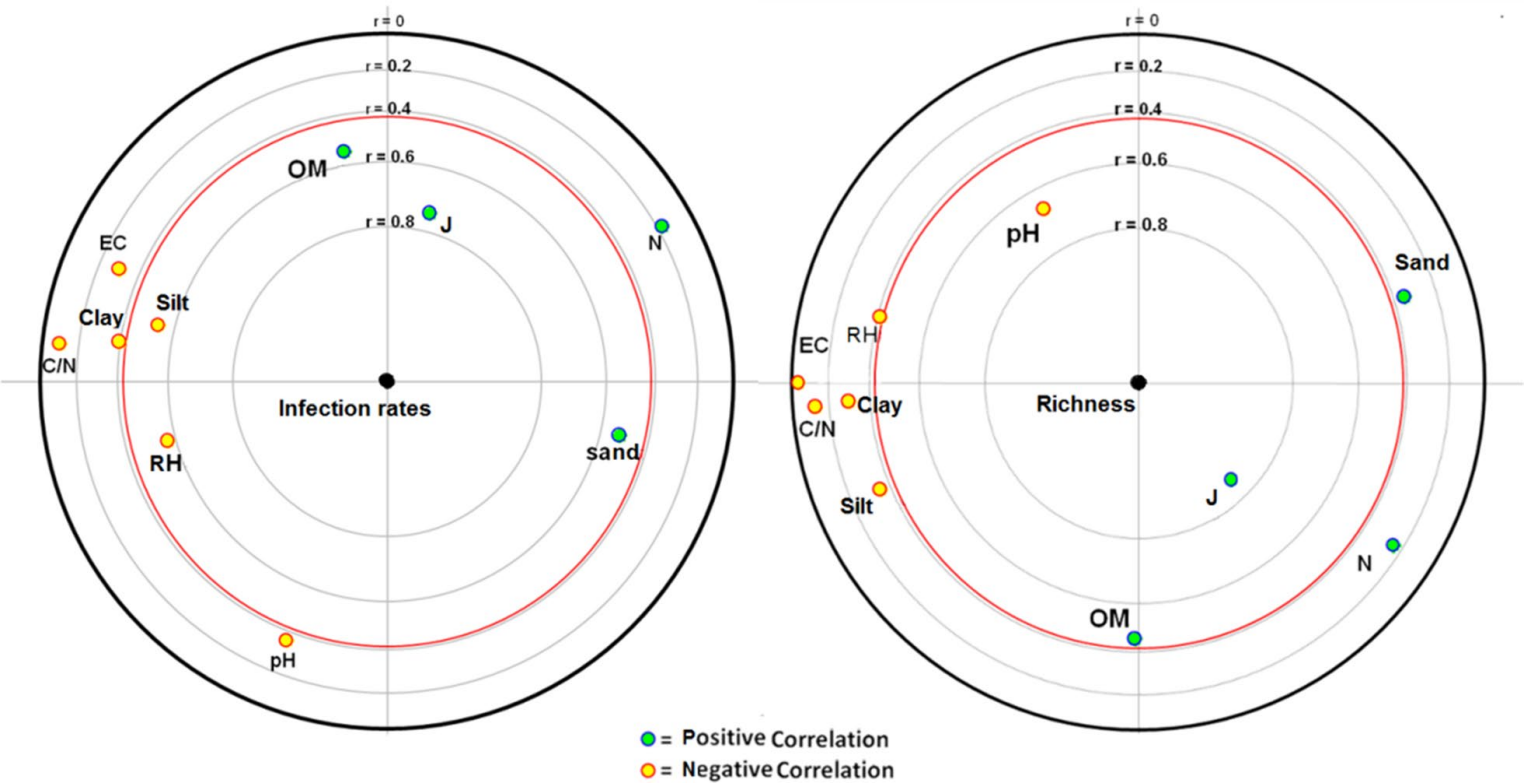
Correlation of the infection percentages and generic richness with the physical and chemical properties of soil namely: texture (sand, silt, and clay), pH, electrical conductivity (EC), humidity (RH), organic matter content (OM) and carbon/nitrogen ratio (C/N)

To illustrate the effect of soil parameters on the generic richness and fungal infection percentages (Ir), we studied the correlation between each parameter to these two factors (Fig. 6). The results demonstrated a strong correlation between infection percentages and soil texture, organic matter content, and evenness. The infection percentages were positively correlated to evenness (J) by more than 70% and approximately 60% to organic matter (OM) and sand content. Relative humidity (RH) and silt content were negatively correlated with the infection percentages by approximately—60%. In general, soil rich in organic matter with adequate humidity was noted to be rich in microorganisms, which increases the probability of insect infection by fungi. High sand content in soil facilitates the mobility of insects as well as fungal conidia. The generic richness of soil was negatively related to pH by − 65% but positively correlated to the evenness (J) by more than 80% and organic matter content (OM) by more than 40%. Moderate pH, high organic matter content, and adequate moisture promote the growth of different fungal species in soil.

### Influence of soil parameters on the distribution of medfly-associated fungi strains

**Fig. 7:**
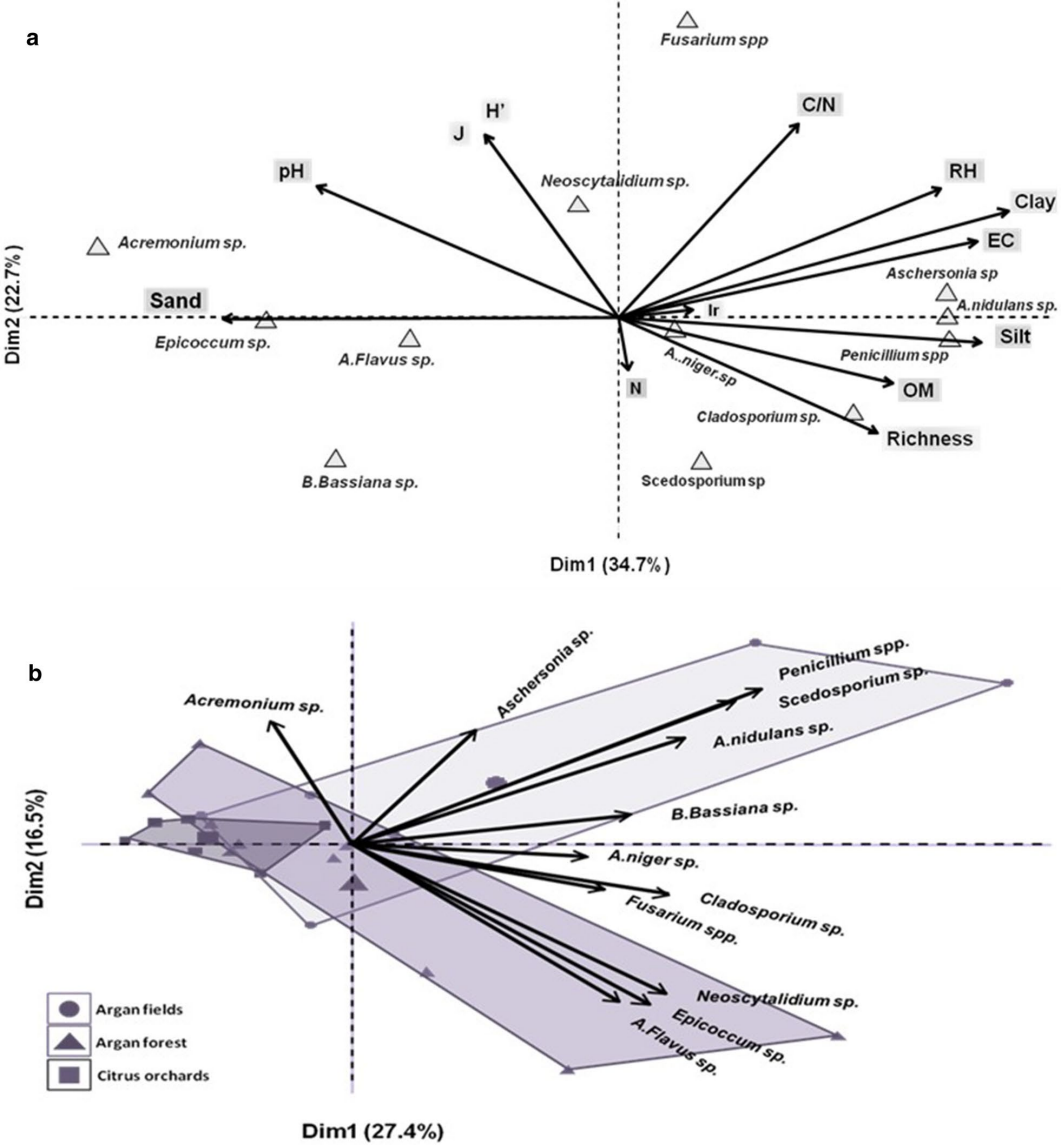
PCA of the effect of soil properties on the distribution of the medfly-associated EPF's genera. **a** effect of physical and chemical properties of soil on the EPF genera distribution, **b** the relationship between soil origin and EPF genera

To understand the relationship between soil parameters and the distribution of EPF strains, an analysis of co-inertia was performed by joining tables of soil parameters and the distribution of strains in the soils. Schematization of these results was carried out in the software “R” by using "Factoextra" and "ggplot2" packages (Fig. 7a). Principal component analysis (PCA) showed that the distribution and abundance of fungi genera are directly influenced by soil health (generic richness, Shannon index, and evenness), pH, C/N, and texture. In general, increased pH and sand content whereas decreased C/N and organic matter content promote the development of highly potent entomopathogenic fungi such as *B. bassiana*, *A. flavus,* and *Acremonium* sp. These species generally grow and develop in the same soils with low generic richness. On the other side, strains of *Aschersonia* sp., *Penicillium* sp., *A. nidulans,* and *Cladosporium* sp. require high relative humidity and very high silt, clay, and organic matter content. Except for *Aschersonia* sp., these species are usually saprophytes and they were better adapted to soils with low generic richness, organic matter content, C/N ratio, and pH as well as toto soils with high relative humidity. Furthermore, the results of this study revealed that there was no effect of soil texture on the availability of species belonging to the genus *Fusarium*.

Besides the physical and chemical properties of soil, results of fungi isolation showed the influence of soil’s origin on the distribution of medfly-associated fungi (Fig. 7b). The classification of strains according to the origins of soils revealed that soils of argan forest and argan fields (argan with crops) are favourable to most of these fungi. In contrast, the soil of citrus orchards was observed to be less suitable for the development of medfly-associated entomopathogenic fungi. The strains of *Aspergillus flavus*, *Epicoccum* sp., *Neoscytalidium* sp., and *Acremonium* sp. were more abundant in forest soils. However, strains of *Penicillium* sp., *Scedosporium* sp., *A. nidulans,* and *Aschersonia* sp. are better adapted to argan fields (argan with crops). The strains of *Fusarium* sp., *A. niger,* and *B. bassiana* were abundant in all soils of argan trees (forest and fields).

In short, the results of this study proved the significant effects of physical and chemical properties of soil and its origin (forest or agricultural soil) on the availability and distribution of medfly-associated entomopathogenic fungi.

## Discussion

Soil is the natural reservoir of entomopathogenic fungi that protects them from abiotic factors (Zimmermann, 1986). Hence, most entomopathogenic fungi are isolated from soil by using either selective media [46, 47] or bait trapping method [26, 27]. To our knowledge, except Imoulan et al. [6, 7] study, entomopathogenic strains against Medfly have never been isolated from soils naturally containing the populations of this insect or from infected individuals. The introduction of non-native entomopathogenic strains can reduce the effectiveness of these biocontrol agents and may pose ecological risks [8, 10]. Thus, by using medfly pupae as bait in its natural environment soils increase the probability to isolate highly virulent indigenous strains that can adapt to the area's environmental conditions [7, 10, 12]. This technique can also determine the diversity of medfly-associated fungi in the studied area.

EPF strains were isolated on PDA medium after trapping with medfly pupae bait. To our knowledge, this is the first report of using *C. capitata* pupae as bait to trap entomopathogenic fungi. During the trapping test, 300 bait pupae (23%) out of 1320 pupae were infected with fungi. Moreover, all of the studied soil samples contained medfly-associated fungi, and pupae infection percentages ranged from 3.33% to over 48% in some samples. Similar results have been reported by Imoulan et al. [6] in Moroccan *Argania spinosa* forests and by Keller et al. [48] in Switzerland, where respectively 91% and 96% of soil samples contained entomopathogenic fungi. However, these rates are very high compared to other studies such as, 71.7% soil samples in Spain [19, 49], 55.5% in China [20], 43% in southern Italy [50], 33.6% in Palestine [51], 20.59% in Turkey [12] and 17.5% in UK [27] contained entomopathogenic fungi. These comparisons must be made cautiously due to the differences in bait species, number of individuals, and the number of soil samples.

Identification of isolated medfly-associated fungi revealed that the most common strains belonged to the genus *Fusarium* (32%) with 83 isolates followed by *Beauveria* (19.23%) with 50 isolates, *Penicillium*, *Cladosporium,* and *Scedosporium* with frequencies over 8%. High occurrence rates of these genera have been reported in soils of Italy, Palestine, and China (Tarasco et al., 1997, Ali-Shtayeh et al., 2003, Sun et al., 2008). Species of these genera, particularly of genus *Fusarium* and *Beauveria* exhibit a wide variety of life strategies including associations with insects and plants [32, 52]. On the other hand, our results showed that strains of *Acremonium* sp., *Aschersonia* sp., *Epicoccum* sp., and *Aspergillus* sp. were less abundant in argan and citrus soils with frequencies less than 6%. Several studies have reported most of these species as entomopathogens [51, 53–55]; while others are classified as opportunistic pathogens [20, 32]. The virulence of these species against the medfly was proved during pathogenicity tests. The present study demonstrates that entomopathogenic fungi are common inhabitants in the soils of the Souss region. Hence, results of this study confirm the findings of Imoulan et al. [6] in Moroccan argan endemic forest; nevertheless, the diversity of the species observed during this study (10 fungal genera) was greater than obtained by Imoulan (only 2 genera: *Beauveria* and *Paecilomyces* (Bainier) (Eurotiales, Thermoascaceae). This difference can be explained by the fact that Imoulan et al. [6] used *Galleria mellonella* (L.) (Lepidoptera: Pyrallidae) larvae as bait and selective media for isolation whereas we used a general medium (PDA) and *C. capitata* pupae as bait.

Principal component analysis (PCA) demonstrated that soil factors are directly correlated to the fungal abundance and infection of pupae. In general, PCA showed that generic richness and ecological balance of soils enhance the chances of fungal infection in insects. Moreover, high sand content and low silt and clay content in the soil favour fungal abundance and infection process. The effect of soil texture on the abundance and availability of entomopathogenic fungi has been reported by several authors. Quesada-Moraga et al. [19] demonstrated that soil texture particularly clay content directly influences the abundance and viability of *B. Bassiana* conidia. They also suggested that high soil clay content improves the abundance and persistence of many entomopathogenic fungi as conidia are adsorbed onto clay particles. Furthermore, Garrido-Jurado et al. [18] proved that the movement of *B. bassiana* and *M. anisopliae* conidia are directly influenced by the soil texture and, adequate sand content promotes the mobility as well as infection percentages of the medfly pupae. On the contrary, excessive sand reduces fungal inoculum due to water drainage. The results also showed that the abundance of entomopathogenic fungi in soil requires a high organic matter content and moderate pH (around 8). Similar results have been reported by previous studies which revealed that entomopathogenic fungi are more abundant in soils with high organic matter content and pH around 8 [14, 19, 48]. This can be explained by the fact that soils with high organic matter content have an ecological balance with a low C/N ratio and a large diversity of arthropod and hosts on which entomopathogenic fungi can grow [10]. However, Meyling and Eilenberg [56] showed that high organic matter content influences antagonistic effect and biological activity in the soil, which negatively affects entomopathogenic species of *Beauveria* and *Metarhizium*. It has also been shown that pH can influence the toxin production of entomopathogenic fungi [19, 57]. The response of fungi to these parameters varies among species. Our results also demonstrated that a high level of relative humidity negatively affects the abundance of entomopathogenic fungi and the rate of pupae infection. This can be explained by the leaching of inoculum and the fast development of saprophytic genera. It is known that the leaching of inoculum is correlated with the amount of water and soil texture [58]. High relative humidity improves the development of saprophytic fungi and competition over space and nutrients [56].

PCA analysis showed that the soils of argan fields and forests are more suitable for the development of medfly-associated fungi compared to citrus orchards soils. Similarly, Tarasco et al. [50] reported that entomopathogenic fungi are more abundant in uncultivated and forest soils. In general, citrus and agricultural soils may contain pesticides that produce a high C/N ratio and can prevent the growth of microorganisms. In addition to silty texture, citrus soils have high pH and ionic charge as well as a high relative humidity due to irrigation, which affects the development of entomopathogenic fungi [18, 19]. This environment may also improve the invasion of soil by saprophytic microorganisms that increase the competition for nutrients and space. Contrarily, soils of argan fields and forests do not generally contain chemical residues and are more suitable for microbial growth by maintaining the ecological balance between these organisms. Moderate pH, good organic matter content, adequate humidity, silty-sandy texture, and absence of toxic pesticides in these soils constitute a good habitat for medfly-associated entomopathogenic fungi. Entomopathogenic fungi also act as endophytes in the absence of a host that explains their high abundance in the soils of argan fields having vegetation throughout the year [11, 59, 60]. On the other hand, recently Uzman et al. [21] have reported that entomopathogenic fungi can even persist in intensively managed vineyard systems. They reported that the application of chemicals had no significant effect on the presence of entomopathogenic fungi in the vineyard soils of Germany. Hence, this resistance of entomopathogenic fungi to fungicides can be exploited for the biological control of pests even in intensively managed agricultural systems. The present study demonstrated the capacity of non-agricultural soils (argan forest in this case) to sustain the development of entomopathogenic fungi against insects. Increasing agricultural practices have reduced such characteristics of the soils. Therefore, securing natural habitats, managing agricultural practices and organic matter, and reducing pesticide applications can improve the conditions for entomopathogenic fungi and natural-enemies. These conditions enhance biological interactions for medfly management and create better sustainable agricultural systems.

Pathogenicity tests showed high virulence of the studied fungal isolates against *C. capitata* pupae, with insect mortality up to 100% for some isolates. This effectiveness could be explained by their origin (Argan forest soil and nearby area, the natural environment of the medfly) or isolation method which used *C. capitata* pupae as a bait. These two parameters probably increased the chance to isolate highly virulent pathogens against medfly. Previous studies have proved that the ability of entomopathogenic fungi to tolerate extreme environmental factors in the field (UV-radiation, water stress, and temperature variation) is correlated with the natural habitat and the origin of these microorganisms [6, 14, 17]. Accordingly, the development of an effective mycoinsecticide requires the selection of highly virulent isolates with high tolerance to the climatic conditions of the application area. Thus, the use of native isolates is preferable due to their adaptation to the climatic conditions of the region [10]. Hence, indigenous isolates could facilitate to effectively control medfly in Morocco and the Mediterranean region due to their ability to tolerate abiotic-stress factors. It will lead to reducing the environmental and ecological risks. However, understanding the ecology of entomopathogenic fungi and assessment of their insecticidal efficiency under controlled conditions for host range, and safety to crop plants is required before their large-scale applications as biological control agents.

During the present study, about 10 genera and 22 different species of medfly-associated entomopathogenic fungi were isolated from different types of soil samples from the Souss region of Morocco. Results confirmed the presence of entomopathogenic fungi in all soil samples. The genera of *Fusarium* (32%) and *Beauveria* (19.2%) were the most abundant. The abundance of these strains was directly affected by the physical and chemical properties of soil such as texture, pH, C/N, and organic matter content as well as soil origin. These indigenous strains are a promising option to effectively control *C. capitata* using bio-control agents. In addition, these results can be useful to determine the suitable soils for applying entomopathogenic fungi against medfly and for the selection of the best adapted fungal species in a particular soil. However, further explorations are needed to select efficient and field-resistant strains.

## Supplementary information

**Additional file 1.** Additional figures.

## Data Availability

The datasets used during and/or analyzed during the current study are available from the corresponding author upon reasonable request.

## Abbreviations

EC: Electrical conductivity
pH: Power of Hydrogen
RH: Relative humidity
OM: Organic matter content
C/N: Carbon/nitrogen ratio
Ir: Infection percentages
R: Species richness
H’: Shannon–wiener index
J: Evenness of fungal communities
CM: Corrected mortality rates
