# Supplementary material for: Diversity of entomopathogenic fungi associated with Mediterranean fruit fly (Ceratitis capitata (Diptera: Tephritidae)) in Moroccan Argan forests and nearby area: impact of soil factors on their distribution
Source: BMC Ecol. 2020 Nov 24;20:64. doi: 10.1186/s12898-020-00334-2 (PMC7684748; doi:10.1186/s12898-020-00334-2)
Supplement: Supplementary file 1 — Additional file 1. Additional figures. [file 12898_2020_334_MOESM1_ESM.docx]

**Appendix: Cultural and morphological characteristics of the mean entomopathogenic fungi genera isolated from *Argania* *spinosa* soils and nearby area using *C. capitata* pupae as bait**


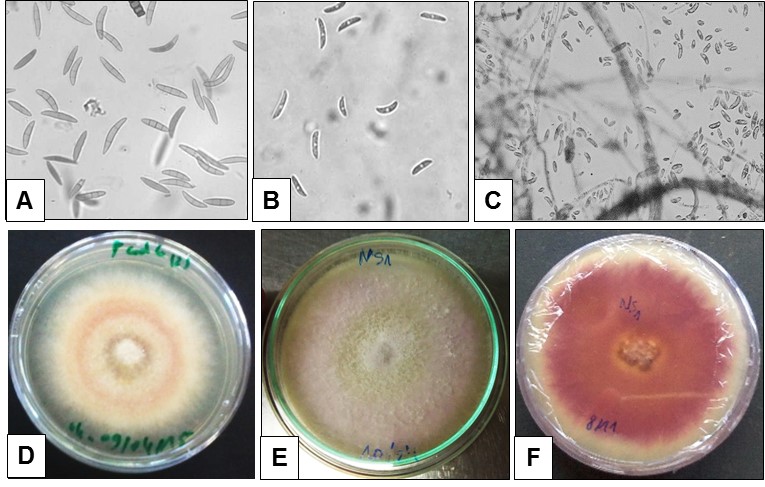


**Fig.S1: Cultural and microscopic morphological characteristics of Fusarium sp. isolates. A, B: Macroconidia and microconidia observed at x1000 magnification; C: Conidia and observed at x400 magnification; D, E, F: colonies aspects on PDA (E and F are representing the F. oxysporum NS1 isolate).**


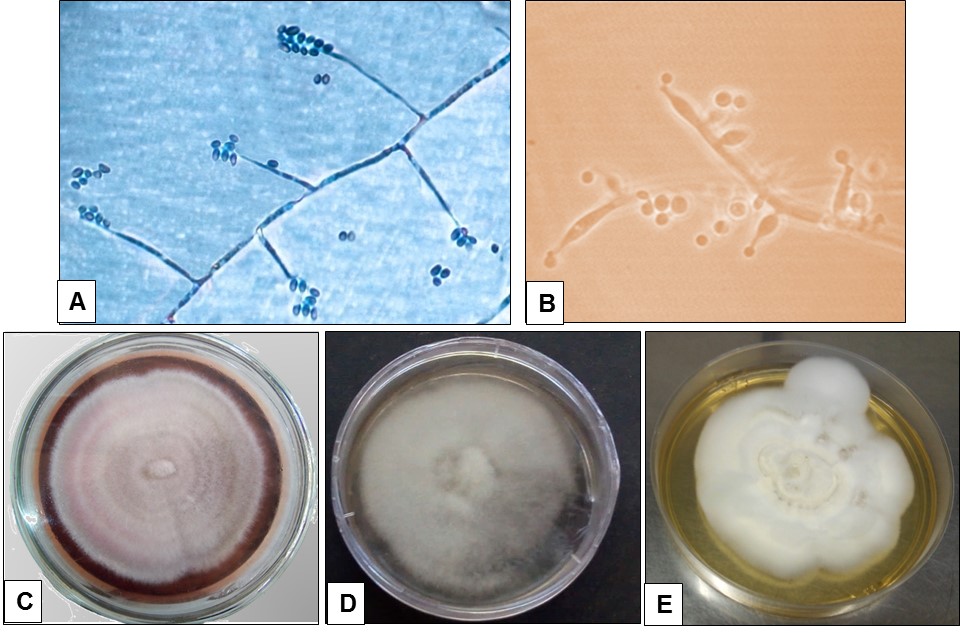


**Fig S2: Cultural and microscopic morphological characteristics of Beauveria bassiana. A, B: Hypha and conidia observed at x400 and x1000 magnification, respectively; C, D, E: colonies aspects on PDA.**


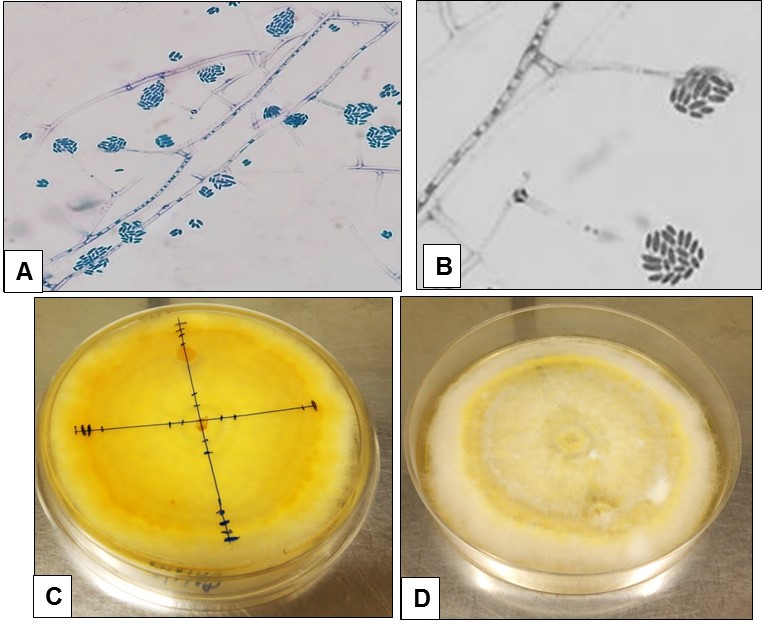


**Fig. S3: Cultural and microscopic morphological characteristics of Acremonium sp. Pt34 isolate. A, B: Hypha and conidia observed at x400 and x1000 magnification, respectively; C, D: colony aspect on PDA.**


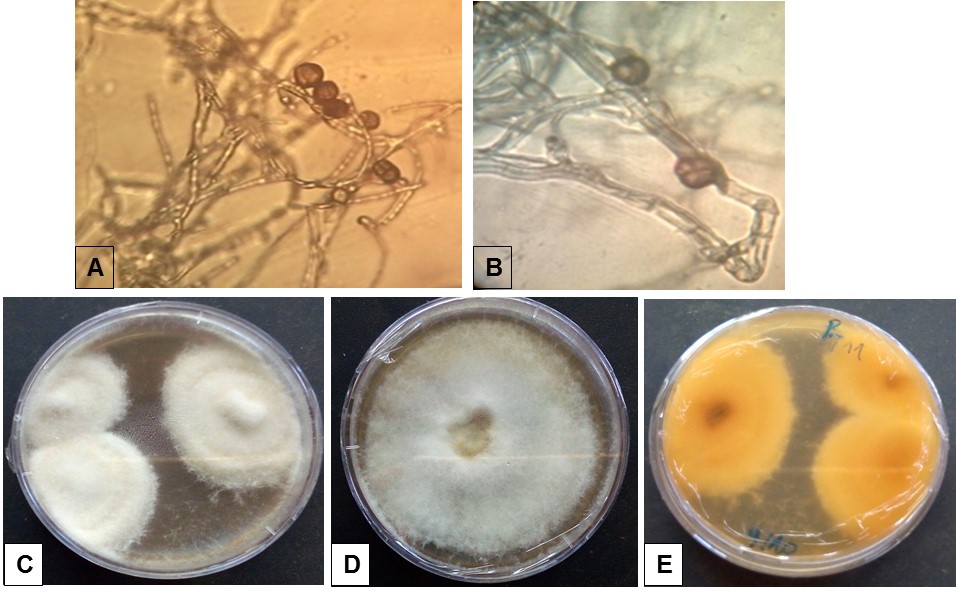


**Fig. S4: Cultural and microscopic morphological characteristics of Epicoccum sp. isolates. A, B: Hypha and conidia observed at x400 and x1000 magnification, respectively; C, D, E: colonies aspects on PDA.**


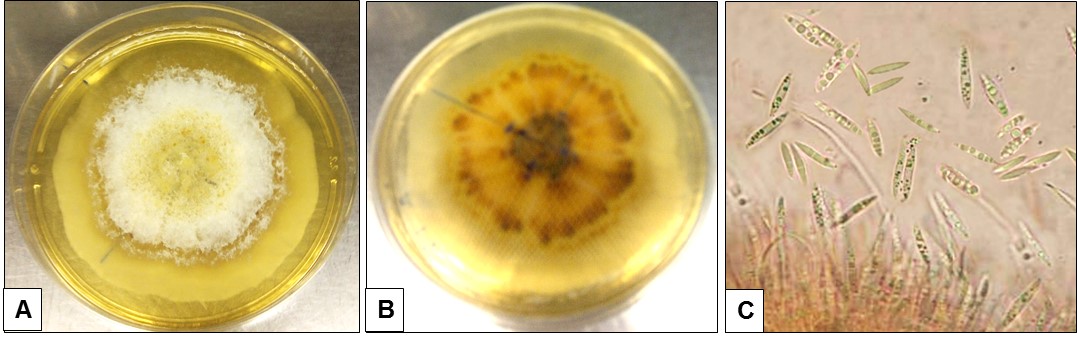


**Fig. S5:** **Cultural and microscopic morphological characteristics of Aschersonia sp. Pt14 isolate. A, B: colony aspect on PDA; C: Macroconidia observed at x1000 magnification.**


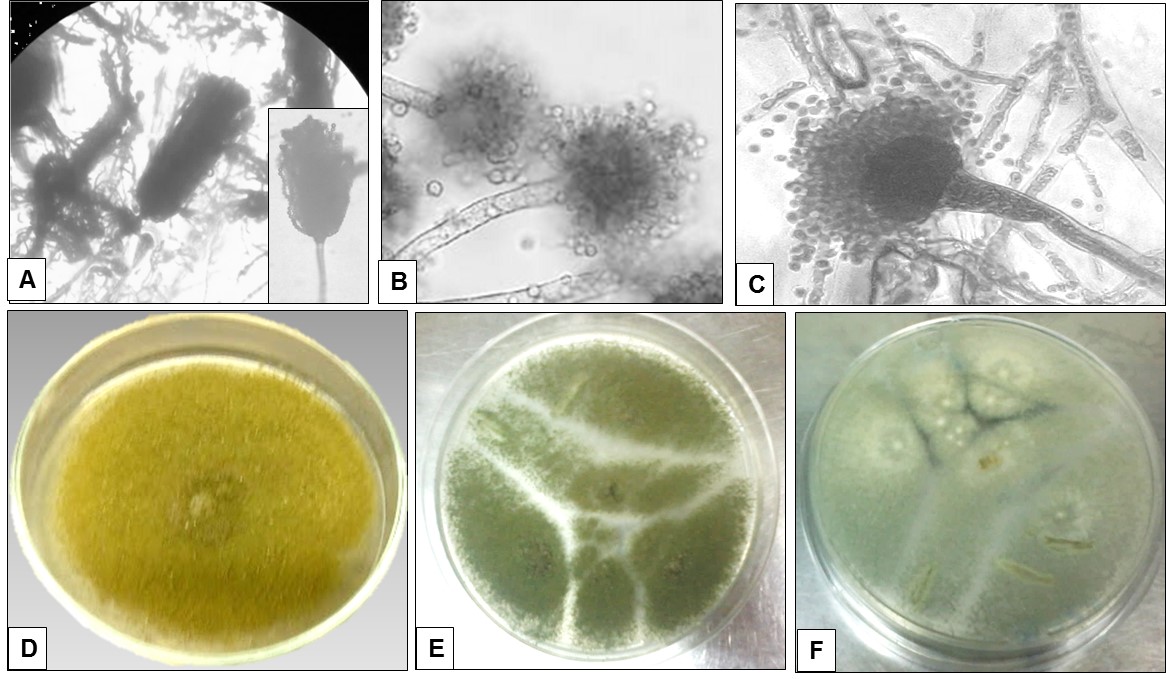


**Fig. S6: Cultural and morphological characteristics of Aspergillus flavus isolates. A, B, C: hypha conidiophores and conidia at x1000 magnification (A shows A. flavus NS14 isolate conidiophore); D, E, F: colonies aspects on PDA.**


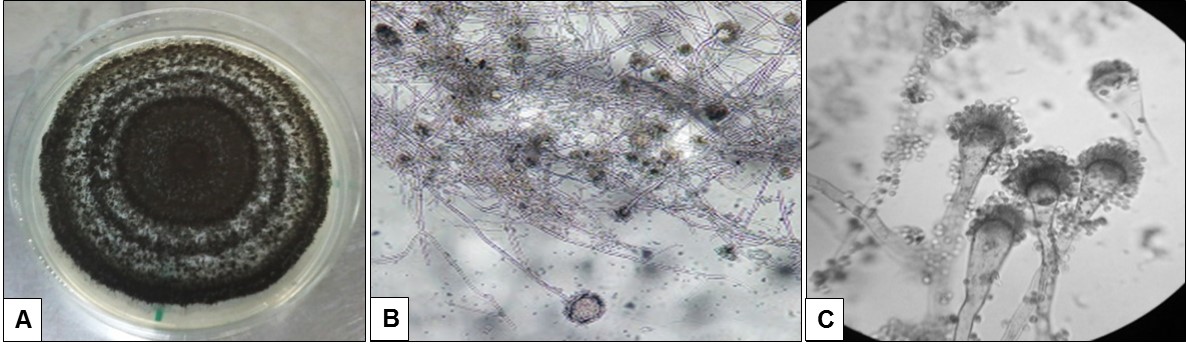


**Fig. S7: Cultural and morphological characteristics of Aspergillus niger. A: colony aspect on PDA; B: hypha conidiophores and conidia at x400 magnification C: conidiophores and conidia at x1000 magnification.**


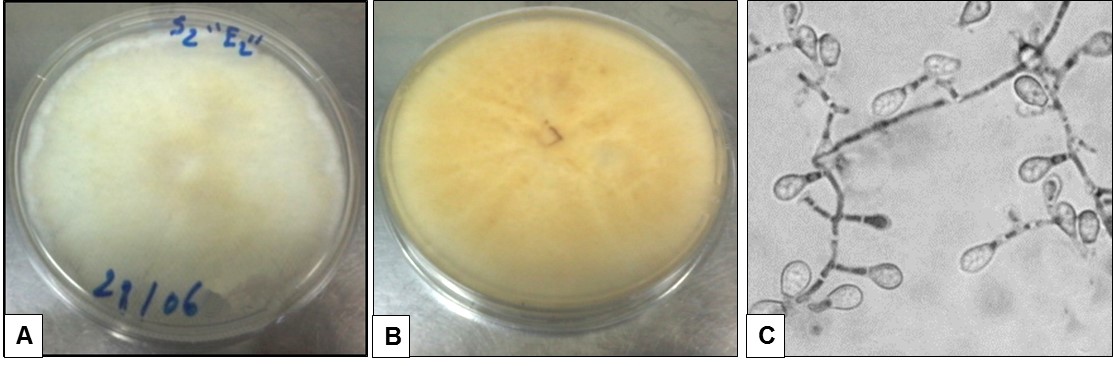


**Fig. S8: Cultural and microscopic morphological characteristics of Scedosporium sp. A, B: colony aspect on PDA; C: Hypha and conidia observed at x1000 magnification.**


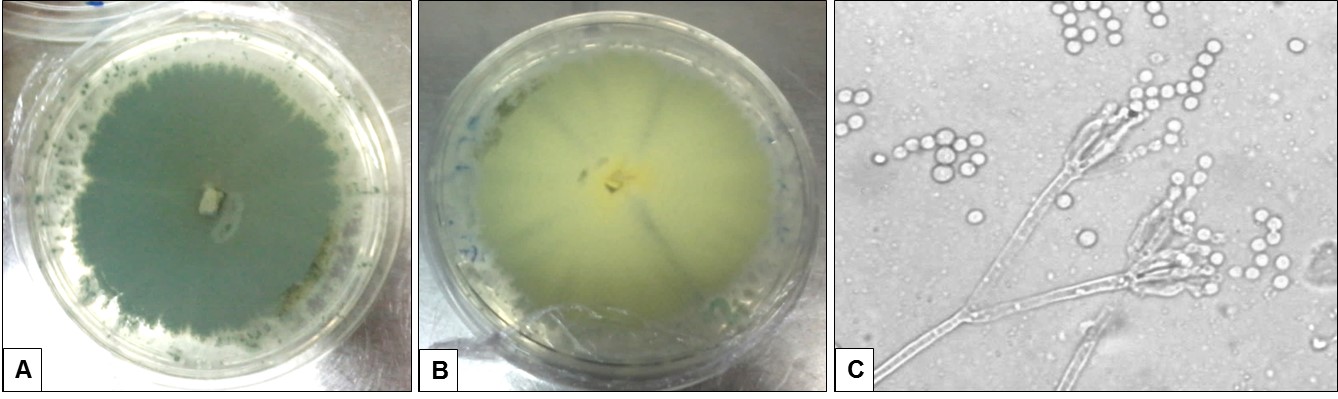


**Fig. S9: Cultural and microscopic morphological characteristics of Penicillium sp. A, B: colony aspect on PDA; C: Hypha and conidia observed at x1000 magnification.**


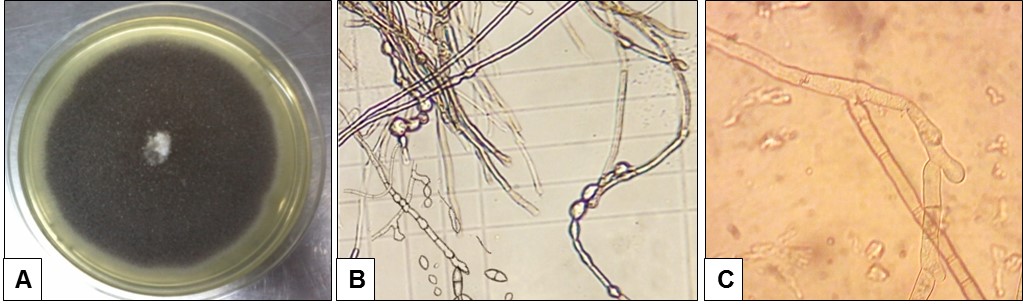


**Fig. S10: Cultural and microscopic morphological characteristics of Cladosporium sp. A: colony aspect on PDA; B, C: Hypha and conidia observed at x400 and x1000 magnification, respectively.**
